# Rapid Identification of Patients Eligible for Direct Emergent Computed Tomography Angiography during Acute Ischemic Stroke: The DARE-PACE Assessment

**DOI:** 10.3390/diagnostics12020511

**Published:** 2022-02-16

**Authors:** Giou-Teng Yiang, Yun-Hao Chen, Pei-Ya Chen, Cheng-Lun Hsiao, Shinn-Kuang Lin

**Affiliations:** 1 Department of Emergency Medicine, Taipei Tzu Chi Hospital, Buddhist Tzu Chi Medical Foundation, New Taipei City 23142, Taiwan; gtyiang@gmail.com (G.-T.Y.); happybrian2@gmail.com (Y.-H.C.); 2 School of Medicine, Tzu Chi University, Hualien 97004, Taiwan; ruentw@gmail.com; 3 Stroke Center, Department of Neurology, Taipei Tzu Chi Hospital, Buddhist Tzu Chi Medical Foundation, New Taipei City 23142, Taiwan; shb@ms19.hinet.net

**Keywords:** acute ischemic stroke, computed tomography angiography, DARE-PACE assessment, endovascular thrombectomy, NIHSS

## Abstract

Background: We investigated the clinical signs to establish a method for rapid identification of patients with the National Institute of Health Stroke Scale (NIHSS) score ≥ 8 eligible for direct brain CTA study; Methods: We retrospectively enrolled 2895 in patients with acute ischemic stroke (AIS). Four items in the NIHSS were selected as the main clinical signs of stroke; Results: A total of 922 (31.8%) patients had an initial NIHSS score of ≥8. The average door-to-CT time and door-to-CTA time were 13.4 ± 1.8 and 75.5 ± 44.5 min, respectively. Among 658 patients who had the priority signs, namely dense hemiplegia (D), aphasia with right arm drop (AR), and eyeball forced deviation (E), 634 patients (96.4%) with an NIHSS score ≥ 8 were identified. By using a classification and regression tree analysis, 153 patients with an NIHSS ≥ 8 were identified among 175 patients (87.4%) who had the secondary signs, namely hemiparesis with limb falls (P), aphasia (A), drowsy or worse consciousness (C), and eyeball limitation (E). The sensitivity, specificity, and accuracy were 85.4%, 97.7%, and 95.3%, respectively. Conclusions: The DARE-PACE assessment involving a checkbox list provides excellent accuracy for rapid identification of AIS patients with an NIHSS score ≥ 8 for direct CTA study to reduce the time delay for endovascular thrombectomy.

## 1. Introduction

Intravenous thrombolysis (IVT) is the most effective pharmacological treatment in the hyperacute stage to improve outcomes of patients with ischemic stroke [1]. Endovascular thrombectomy (EVT) is a direct intra-arterial approach to removing the thrombus in the occluded artery and enables complete patency of the artery with effective recanalization [2]. However, the efficacy of IVT and EVT decreases with time elapsed from symptom onset. The recommended therapeutic time window is within 3–4.5 h for IVT and within 6 h for initiation of EVT in anterior circulation large vessel occlusion (LVO) after symptom onset [3,4,5]. Most studies have found that bridging therapy, combining IVT and subsequent EVT, still provides more beneficial effects than EVT alone [6,7]. The current American and European guidelines recommend IVT therapy for patients with LVO before EVT [4,8]. A common pathway for rapid IVT treatment of acute ischemic stroke (AIS) involves the immediate noncontrast computed tomography (NCCT) to save time at the ED. The decision regarding EVT depends on the results of a further cerebral angiography. However, there is usually a time delay between the first NCCT and the subsequent CTA.

The American Heart Association and American Stroke Association recommend a National Institute of Health Stroke Scale (NIHSS) score of ≥6 for EVT.4. In Taiwan, the NIHSS score for EVT recommended by the National Health Insurance Administration ranges between 8 and 30 [9]. Based on this criterion, patients who presented to the ED with a suspected acute stroke with an NIHSS score of ≥8 were eligible for a brain CTA study and possible further EVT. Previous studies have investigated the correlations among different stroke scales, cutoff levels of NIHSS score, and LVO [10,11,12,13,14]. The association between most scales, either applied at the prehospital or ED setting, and LVO has been determined. While it is impractical in clinical settings, in real-world scenarios at the ED, screening a patient for a CTA study as early as possible to detect a possible LVO that warrants further EVT treatment is more crucial than accurately detecting a patient with LVO.

In some comprehensive stroke centers, instead of an NCCT, a direct emergent brain CTA study has been conducted in patients with suspected AIS for early detection of LVO. Nevertheless, the benefits of an emergent CTA study and further EVT for patients with a minor degree of stroke, with an NIHSS score < 6, remain controversial [4]. A contrast-enhanced brain CTA study requires at least 5 min longer than an NCCT, which causes a delay in IVT. Moreover, the majority of patients presented to the ED had a minor degree of severity with an NIHSS score < 5, in whom a CTA study and EVT treatment are usually not indicated. Unnecessary exposure of patients to the contrast medium, as well as squandering of medical resources for patients with a minor degree of stroke, makes the application of a routine brain CTA study in all patients challenging. A simple convenient method for efficiently identifying eligible patients for a direct CTA study within a short time is crucial in reducing time delay. We studied the correlation between clinical signs and an NIHSS score and established a simple assessment method for the rapid identification of AIS patients eligible for a direct brain CTA study.

## 2. Patients and Methods

### 2.1. Stroke Population and Data Collection

The stroke registry database was retrospectively reviewed to identify patients who received treatment for AIS in a neurological ward from May 2010 to May 2019. Information on the initial NIHSS score on arrival at the ED was collected. Since 2018, the studied hospital established EVT treatment with a 24-h availability performed by neuroradiologists based on contrast brain CTA. Door-to-CT time and door-to-CTA time were obtained for patients who fulfilled the IVT or EVT criteria.

### 2.2. Statement of Ethic

The study was conducted per the Declaration of Helsinki. Ethical approval for this study was provided by the Institutional Review Board of Taipei Tzu Chi Hospital, New Taipei City (approval no. 10-XD-040). Informed written consent was waived because the study was retrospective in nature.

### 2.3. Identification of Stroke Severity and IVT or EVT Protocol

Code stroke is activated by the emergency physician when a patient is eligible for evaluation of IVT or EVT with the presence of any of the three components of the Cincinnati Prehospital Stroke Scale (arm weakness, speech, or facial droop) [15]. The standard procedure included an immediate NCCT scan and an emergency consultation with the on-duty neurologist. Stroke severity was assessed using the NIHSS, usually after an NCCT study by neurology consultants. We followed the treatment guideline of the Taiwan Stroke Society and the reimbursement criteria of the National Health Insurance Administration for EVT, which was an NIHSS score between 8 and 30 [9,16]. Intravenous thrombolysis with a loading dose of alteplase is initiated, followed by a subsequent brain CTA during infusion the remaining dose of alteplase if the patient had an NIHSS score ≥ 8.

### 2.4. Decision Regarding Main Signs for Rapid Identification of Patients Eligible for Thrombectomy

Four items included in the NIHSS, namely item 1a level of consciousness, item 2 best gaze, items 5a6a motor arm and leg (left) together with 5b6b motor arm and leg (right), and item 9 best language, were selected as the main clinical signs of stroke to rapidly identify patients eligible for a direct CTA study. Item 1a is scored as follows: 0, alert; 1, drowsy; 2, stuporous; and 3, coma. Item 2 is scored as follows: 0, normal; 1, partial gaze palsy; and 2, forced deviation. Items 5 and 6 are scored as follows: 0, no drift; 1, drift; 2, cannot resist gravity; 3, no effort against gravity; and 4, no movement. Item 9 is scored as follows: 0, no aphasia; 1, mild to moderate aphasia; and 2, severe aphasia. The three most important signs implicating severe stroke with a high possibility of an NIHSS score ≥ 8 were designated as priority signs, namely dense hemiplegia (item 5a + 6b or 5b + 6b ≥ 6; unable to raise both arm and leg against gravity on the same side), aphasia (item 9 ≥ 1; any type of aphasia) with right arm drop (item 5b ≥ 3; unable to raise the right arm against gravity), and eyeball forced deviation (item 2 = 2). If patients did not present the above priority signs, further secondary signs could be assessed using the same items with optimal cutoff levels. In such a situation, items 5a + 6a (left arm and left leg) or items 5b + 6b (right arm and right leg) were assessed together. For instance, if a patient had right hemiparesis with a score of 3 for item 5b (right arm) and a score of 1 for item 6b (right leg), the total score for item 5 + 6 would be 4.

### 2.5. Statistical Analysis

Continuous variables are presented as the mean ± standard deviation. The chi-square test and Fisher’s exact test were used for comparisons of categorical variables. Each of the four selected NIHSS items was converted into dichotomous variables, with the optimal cutoff levels determined according to the Youden index by using the receiver operating characteristic (ROC) curve for the NIHSS score ≥ 8. The variables were added to a multiple logistic regression model to identify the significance associated with an NIHSS score ≥ 8. We compared the predictive performance of the variables by using the C-statistic for an NIHSS score ≥ 8. In addition, the classification and regression tree (CART) analysis was used to determine and stratify patients by best splitting rules according to the important signs for an NIHSS score ≥ 8. A terminal node containing >75% of patients with an NIHSS score ≥ 8 indicated effective classification, and the numbers of patients with an NIHSS score ≥ 8 were recorded as successful selections. A *p*-value of <0.05 was considered to indicate a significant result. All statistical analyses were performed using SPSS (version 24; SPSS Inc., Chicago, IL, USA). The ROC curves were compared using MedCalc version 18 (MedCalc Software, Mariakerke, Belgium).

## 3. Results

In total, 2895 patients were enrolled in the study. Among those, 922 (31.8%) patients had an initial NIHSS score ≥ 8. The average liaison-to-neurological evaluation time for a code stroke was only 6 min. Both NCCT and CTA were performed at the ED for possible EVT in 111 patients. The average door-to-CT time and door-to-CTA time were 13.4 ± 1.8 and 75.5 ± 44.5 min, respectively.

For the priority signs, 576 of 595 patients (96.8%) with an NIHSS score ≥ 8 had dense hemiplegia (D), 260 of 261 patients (99.6%) with an NIHSS score ≥ 8 had aphasia with right arm drop (AR), and 212 of 218 patients (97.2%) with an NIHSS score ≥ 8 had eyeball forced deviation (E). Overlapping signs were observed in patients with priority signs. We first selected 595 patients with dense hemiplegia, then selected 17 patients who had aphasia with right arm drop, and then further screened 46 patients with eyeball forced deviation. Thus, a total of 634 patients with an NIHSS score ≥ 8 (96.4%) were identified among 658 patients who had priority signs. After screening out 658 patients with priority signs, 288 patients with an NIHSS score ≥ 8 remained among the 2237 patients. Through ROC curve analyses, the optimal cutoff value of an NIHSS score ≥ 8 for item 1a consciousness was ≥1, for item 2 gaze was ≥1, for item 5 + 6 hemiparesis was ≥3, and for item 9 aphasia was ≥1. We translated the cutoff values of each item into secondary simple signs, namely drowsy or worse consciousness (C), hemiparesis with limb falls (P; rapid arm or leg falls from raised position; some effort against gravity), any type of aphasia (A), and eyeball limitation (E). Multivariable logistic regression analysis revealed that all three priority signs and four secondary signs were strong significant predictors of the NIHSS score ≥ 8 (Table 1).

Table 2 presents the C-statistics estimated from the stepwise forward regression models for the detection of an NIHSS score ≥ 8 for each sign. Among the priority signs, dense hemiplegia had the most significant predictive performance with a C-statistic of 0.809. The addition of aphasia with right arm drop and eyeball forced deviation to the regression model resulted in significant stepwise improvements in the C-statistics from 0.809 to 0.839 (*p* < 0.001) in 2895 patients. Similar results were observed for the secondary signs. The addition of aphasia, drowsy or worse consciousness, and eyeball limitation to the regression model of hemiparesis with limb falls resulted in significant stepwise improvements in the C-statistics from 0.728 to 0.923 (*p* < 0.001) in 2237 patients without priority signs.

**Figure 1:**
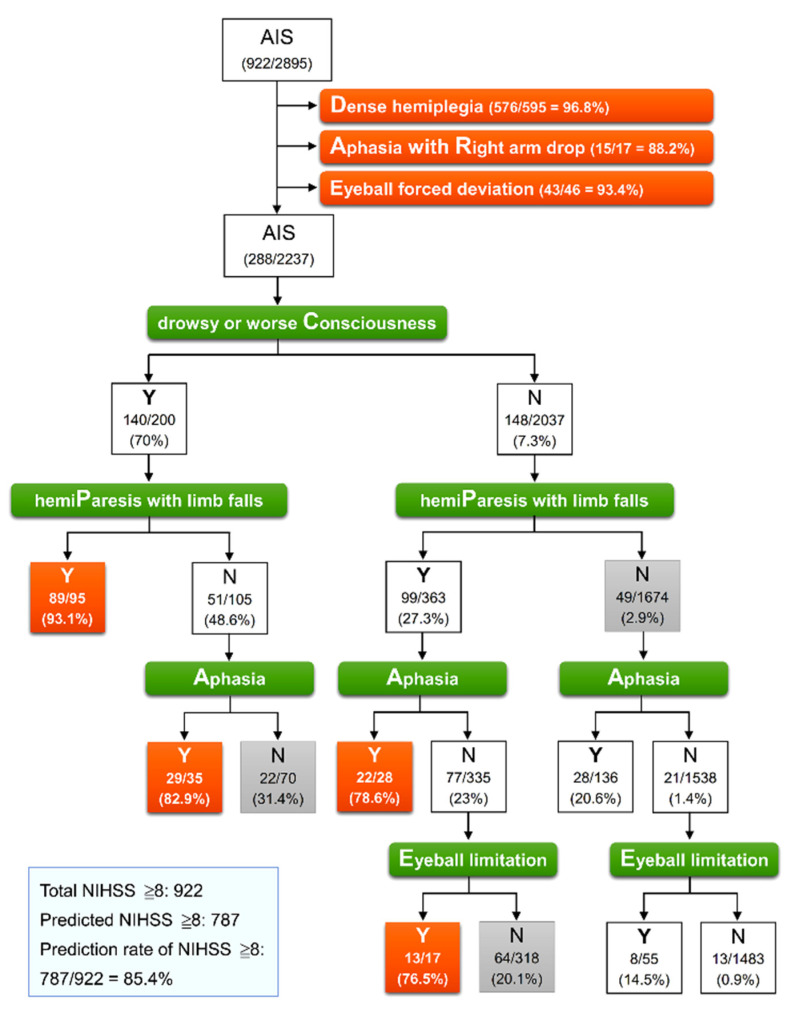
Classification tree model for identification of NIHSS ≥ 8 in 2895 patients with acute ischemic stroke. At each intermediate node, an observation moves to the left child node if and only if the stated condition is true (Y). The pair of numbers inside each terminal node denotes the number classified and the node sample size with its percentage. Orange node indicates that a classification rate of true value exceeds 75%, representing effective classification. AIS, acute ischemic stroke; NIHSS, National Institute of Health Stroke Scale.

We used a CART analysis determined by the abovementioned four secondary signs to investigate the best split rule and to identify an NIHSS score of ≥8 in 2237 patients without priority signs. Figure 1 presents the results of classification trees determined using the priority signs in all 2895 patients, followed by using the secondary signs in 2237 patients. Effective classification was observed in four terminal nodes containing >75% patients with an NIHSS score ≥ 8. The most important factor, the first defining split, among the secondary signs was drowsy or worse consciousness. The subsequent defining split factor was hemiparesis with limb falls. Of 95 patients, 89 (93.1%) with both drowsy or worse consciousness and hemiparesis with limb falls had an NIHSS score of ≥8 (PC). In patients who had drowsy or worse consciousness but no hemiparesis with limb falls, the presence of aphasia enabled the identification of 29 of 35 patients (82.9%) with an NIHSS score of ≥8 (AC). Among the 2037 patients without drowsy or worse consciousness, both hemipareses with limb falls and aphasia (PA) and both hemipareses with limb falls and eyeball limitation (PE) enabled the identification of 22 of 28 patients (78.6%) and 13 of 17 patients (76.5%) with an NIHSS score of ≥8, respectively. Thus, among 2895 patients with AIS, 787 patients with an NIHSS score ≥ 8 could be identified, of which 634 patients were identified through priority signs and a further 153 were identified from four effective terminal nodes through secondary signs, accounting for 85.4% of 922 patients with a true NIHSS score ≥ 8.

This model of classification trees had a sensitivity of 85.4%, specificity of 97.7%, an accuracy of 93.7%, and an area under the ROC curve of 0.915 (0.904–0.925) for identifying patients with an NIHSS score ≥ 8 (Table 3). Given the complexity of this model of classification trees, we simplified and converted the results into a user-friendly and easy-to-remember system: the DARE-PACE assessment (Table 4). An immediate screen with priority signs was able to identify 68.8% of patients with an NIHSS score ≥ 8. If DARE signs were not observed, an additional evaluation of the secondary signs was able to identify a further 16.6% of patients with an NIHSS score ≥ 8. Patients who had any sign of DARE or two signs of PAC (PA, PC, or AC) or PE were candidates for direct CTA study. We applied this DARE-PACE model to test its accuracy for identifying patients with an NIHSS ≥ 6, as recommended by the American guideline. A total of 828 of 1236 patients (70%) who had an NIHSS of ≥6 were identified with a sensitivity of 70%,a specificity of 99.3%, an accuracy of 85.5%, and an area under the ROC curve of 0.832 (Table 3).

## 4. Discussion

This study provided a novel useful assessment model for the rapid identification of patients eligible for direct CTA study and hence shorten in-hospital delay by 62 min. This DARE-PACE system comprises a two-step assessment, namely the priority DARE signs, which could identify 68.8% of patients with an NIHSS score ≥ 8, and the secondary PACE signs, which could identify a further 16.6% of patients with an NIHSS ≥ 8. The 14.6% of patients with an NIHSS score ≥ 8 not identified through this assessment could be evaluated with detail NIHSS through the regular pathway after an NCCT study by neurology consultants, who arrived at the ED within an average of 6 min after a consultation. Furthermore, only 2.3% of patients with an NIHSS score < 8 who did not fulfill the criteria for EVT were selected for a CTA study. However, we were able to prevent 66.6% (1927/2895) of patients with a relatively minor degree of stroke (NIHSS < 8) from an unnecessary emergent CTA study. We also were able to prevent time delay in IVT treatment in case an emergent CTA was regarded a regular study for all patients with AIS in some comprehensive stroke centers [17,18].

Several clinical scales or assessments have been developed to rapidly evaluate acute stroke patients with presumed LVO. The three-item stroke scale comprises a level of consciousness, gaze, and motor function with a score ranging from 0 to 6. A score of ≥4 predicted proximal vessel occlusion as well as an NIHSS of ≥14 [10]. The Los Angeles Motor Scale comprises the facial droop, arm shift, and grip strength components with a score ranging from 0 to 5, and a score of ≥4 was highly associated with a persistent LVO [11]. The Rapid Arterial oCclusion Evaluation scale, comprises facial palsy, arm and leg motor function, head and gaze deviation, aphasia, and agnosia with a score ranging from 0 to 9, and a score of ≥5, had the highest accuracy in predicting LVO [12]. The Cincinnati Prehospital Stroke Severity Scale (CPSSS) comprises conjugate gaze deviation, level of consciousness, and motor arm with a score ranging from 0 to 4, and a score of ≥2 most accurately predicted an NIHSS score of ≥15 and an LVO [13]. The VAN assessment indicates three items (actually four items, including the must-have motor weakness) comprises visual disturbance, aphasia, and neglect with no scoring system [14]. The presence of limb weakness plus one or all of the V, A, or N components identified patients with LVO and an NIHSS score ≥ 6. The scoring system presented above focused on identifying patients with LVO and higher NIHSS scores and was more useful in prehospital evaluation for direct transport to comprehensive stroke centers. Evaluation and interpretation of visual disturbance and neglect, which are subtle signs of stroke, require special training (a 2-h training session for triage nurses to obtain proof in the VAN study), are time-consuming, and are not usually as accurate as those performed by neurologists. Further interobserver reliability in larger patient groups is required to validate the clinical efficacy of the VAN system [14].

The DARE-PACE assessment model developed in this study contains similar evaluation components as the three-item stroke scale and CPSSS (level of consciousness, motor, and gaze evaluation) but has an additional language function and no scoring system. The time required for recognition of the priority DARE signs might not be longer than 1 min. In this study, any type of aphasia (motor, sensory, or global aphasia) was considered aphasia. Patients with motor and global aphasia who are unable to talk can be easily recognized. Aphasia with right arm drop indicates a higher degree of acute stroke in the left hemisphere involving the motor cortex. If no priority DARE signs were observed, physicians proceeded to check for the secondary PACE signs. Patients with impaired consciousness alone may not necessarily present a condition of stroke. Further focal neurological deficits, such as hemiparesis or aphasia, helps recognize a stroke condition. The secondary sign combining hemiparesis with limb falls and aphasia (PA) was able to identify patients who were left-handed with left hemispheric dominance presenting aphasia with left limbs weakness [19]. Completion of the secondary PACE assessment might require 1 min. Given the same assessment items with different levels of severity in DARE and PACE, physicians can evaluate both priority and secondary signs simultaneously within a short time of not more than 2 min. Compared with scoring systems and the VAN assessment, the DARE-PACE assessment provides a more convenient and easier method for emergent physicians or nurses. The preferred pathway for DARE-PACE assessment is attaching a checklist to the medical record (Table 4). Physicians need simply go through the checklist and mark the checkbox if a patient presents the corresponding sign. Patients who receive a checkmark in any of the seven checkboxes are candidates for a direct CTA study.

**Figure 2:**
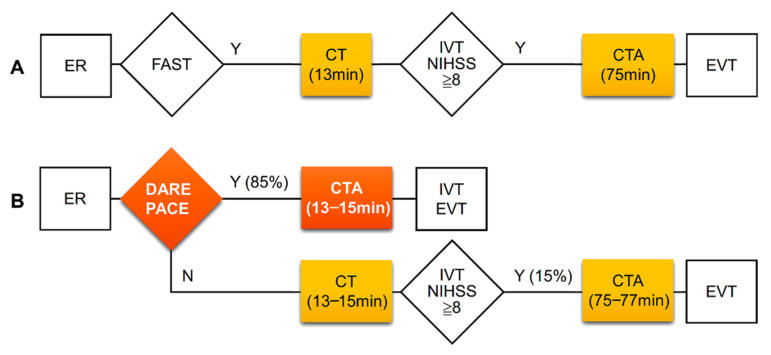
Clinical pathways and time intervals of CT and CTA studies at the ED before (**A**) and expected improvement after (**B**) application of the DARE-PACE assessment. The DARE-PACE assessment (**B**) could identify about 85% of patients with NIHSS ≥ 8 hence shorten in-hospital delay by 62 min. CT, computed tomography; CTA, computed tomography angiography, ED, emergency department; EVT, endovascular thrombectomy; FAST, the Cincinnati Prehospital Stroke Scale (facial droop, arm weakness, and speech disturbance); IVT, intravenous thrombolysis.

The DARE-PACE assessment had excellent accuracy with an area under the curve of 0.915 and a good accuracy with an area under the curve of 0.832 for the identification of patients with an NIHSS score ≥ 8 and with an NIHSS score ≥ 6, respectively. We did not evaluate the accuracy of the DARE-PACE assessment in predicting LVO because this was a retrospective study and the goal of the assessment was to identify patients eligible for the CTA study. Whether LVO was present or not could be confirmed immediately after a CTA study in the selected patients. In the present study, there was a long delay between average door-to-CT time (13 min) and door-to-CTA time (75 min). Through the DARE-PACE assessment, 85% of patients with an NIHSS ≥ 8 could receive a direct CTA study with a reduced door-to-CTA time by 62 min (Figure 2).

Contrast-associated acute kidney injury is a concern. Obtaining serum creatinine levels from an emergent blood test within 13 min after patients’ arrival at the ED before a CTA study is difficult. The risk of acute kidney damage after prolonged exposure to contrast medium has been reported to be very low, with 1–2% of patients with an estimated glomerular filtration rate of >30 mL/min/1.73 m^2^. However, this was increased in patients with an estimated glomerular filtration rate of <30 mL/min/1.73 m^2^ [20,21]. In such a situation, intravenous isotonic volume expansion with normal saline after contrast medium administration is the preferred method to prevent further injury [21]. However, severe life-threatening adverse events occur rarely. A bedside creatinine point-of-care testing, which has been reported to correlate well with central laboratory values, could be a reliable method to be applied at the ED, which allows the rapid screening of creatinine levels within several minutes before a CTA study [22]. A recent study reported by Mayer et al. suggested that performing emergent CTA for all AIS patients presenting within 24 h, regardless of the baseline NIHSS and the renal function, increased the EVT treatment population. This was associated with a trend toward improved outcomes among LVO patients presenting within 6 h of symptom onset [18]. However, as limitations addressed by the authors, certain results remained speculative and tenuous, and data regarding contrast-induced nephropathy as well as cost-effectiveness were lacking.

This study has several limitations. First, this was a retrospective study. A prospective study with the DARE-PACE assessment is required to evaluate its clinical application and interobserver reliability. Second, we did not perform an external validation of the DARE-PACE assessment with other stroke cohorts. Nevertheless, this study provided reliable and accurate results based on a large sample size. Third, we did not stratify patients by anterior and posterior circulation. Some patients with a small area of brainstem infarct might present a dense hemiplegia with an NIHSS > 8. A brain CTA study in such patients may also be necessary because a small area of infarct could be caused by a high-grade stenosis of the basilar artery, which has the potential to progress to total occlusion causing coma.

## 5. Conclusions

The two-step DARE-PACE assessment involving a convenient checkbox list comprising four major clinical signs provides excellent accuracy in the rapid identification of AIS patients with an NIHSS score ≥ 8 at the ED. A direct emergent CTA study in patients identified using the DARE-PACE assessment could substantially reduce the time delay for EVT.

## Figures and Tables

**Table 1:** Multivariable logistic regression analyses of clinical signs for predicting an NIHSS score ≥ 8 in patients with acute ischemic stroke.

Total Patients (*n* = 2895)				Exclusion of Patients with Priority Signs (*n* = 2237)			
Priority Sign	OR	95% CI	*p*	Secondary Sign	OR	95% CI	*p*
Dense hemiplegia	121.052	74.147–197.630	<0.001	Hemiparesis with limb falls	21.773	14.341–33.054	<0.001
Aphasia with right arm drop	113.738	15.112–856.014	<0.001	Aphasia	17.057	10.559–27.555	<0.001
Eyeball forced deviation	45.377	19.113–107.731	<0.001	Drowsy or worse consciousness	24.216	14.727–39.820	<0.001
				Eyeball limitation	11.907	6.481–21.874	<0.001

CI, confidence interval; NIHSS, National Institutes of Health Stroke Scale; OR, odds ratio.

**Table 2:** C-statistic of priority signs and secondary signs for predicting NIHSS ≥ 8 in patients with acute ischemic stroke.

Total Patients (*n* = 2895)				Exclusion of Patients with Priority Signs (*n* = 2237)			
Priority Sign	C	95% CI	*p*	Secondary Sign	C	95% CI	*p*
Dense hemiplegia	0.809	0.789–0.829		Drowsy or worse consciousness	0.728	0.690–0.765	
Includes eyeball forced deviation	0.830	0.811–0.850	<0.001	Includes hemiparesis with limb falls	0.863	0.836–0.891	<0.001
Further includes aphasia with right arm drop	0.839	0.821–0.858	<0.001	Further includes aphasia	0.907	0.886–0.927	<0.001
				Further includes eyeball limitation	0.923	0.906–0.941	<0.001

CI, confidence interval; NIHSS, National Institutes of Health Stroke Scale.

**Table 3:** Diagnostic accuracy of classification trees for NIHSS ≥ 8 and NIHSS ≥ 6.

	NIHSS ≥ 8	NIHSS < 8	Total		NIHSS ≥ 6	NIHSS < 6	Total
Predicted NIHSS ≥ 8	787	46	833	Predicted NIHSS ≥ 6	828	11	839
Predicted NIHSS < 8	135	1927	2062	Predicted NIHSS < 6	408	1648	2056
Total	922	1973	2895	Total	1236	1659	2895
Prevalence of NIHSS ≥8: 31.8% Sensitivity: 85.4% Specificity: 97.7% Accuracy: 93.7% Positive predictive value: 94.5% Negative predictive value: 93.5% Positive likelihood ratio: 36.61 Negative likelihood ratio: 0.15 Area under ROC curve: 0.915 (0.904–0.925)				Prevalence of NIHSS ≥6: 42.7% Sensitivity: 70.0% Specificity: 99.3% Accuracy: 85.5% Positive predictive value: 98.7% Negative predictive value:80.2% Positive likelihood ratio: 101.03 Negative likelihood ratio: 0.33 Area under ROC curve: 0.832 (0.817–0.845)			

NIHSS, National Institutes of Health Stroke Scale.

**Table 4:** Criteria for direct emergent brain CTA (NIHSS score ≥ 8).

Criteria for Direct Emergent Brain CTA (NIHSS ≥ 8): DARE-PACE
Priority signs (Any sign of **DARE**): □ (1) **D**ense hemiplegia (unable to raise arm and leg against gravity) □ (2) **A**phasia with **R**ight arm drop (Any type of aphasia + unable to raise right arm against gravity) □ (3) **E**yeball forced deviation Secondary signs (Any two signs of **PACE: PA, PC, PE, or AC**): (1) Hemi**P**aresis with limb falls (rapid arm or leg falls from raised position) □ **P** + **A**phasia (any type of aphasia) □ **P** + drowsy or worse **C**onsciousness □ **P** + **E**yeball limitation □ (2) **A**phasia + drowsy or worse **C**onsciousness

Patients who obtain a checkmark in any of seven checkboxes are candidates for direct CTA study (sensitivity: 85.4%, specificity: 97.7%, accuracy: 93.7%). CTA, computed tomography angiography; NIHSS, National Institutes of Health Stroke Scale.

## Data Availability

The data presented in this study are available on request from the corresponding author.
